# From inherited susceptibility to tumor state: toward a stage-aware architecture for genomic risk stratification in prostate cancer

**DOI:** 10.3389/fgene.2026.1969511

**Published:** 2026-10-01

**Authors:** Ziheng Zheng, Xuechun Yang, Minqian Ou, Yuedong Chen, Deyong Gan

**Affiliations:** 1 The First Clinical College of Chongqing Medical University, Chongqing, China; 2 Department of Gastrointestinal Surgery, Tongliang Hospital of the First Affiliated Hospital of Chongqing Medical University, Chongqing, China

**Keywords:** artificial intelligence, genomic classifier, multi-omics integration, polygenic risk score, prostate cancer, risk stratification, transcriptomics

## Abstract

Prostate cancer risk is currently represented by two largely separate genomic layers. Germline polygenic scores estimate inherited susceptibility before diagnosis, whereas tumor genomic and transcriptomic classifiers estimate biological aggressiveness and treatment response after tissue acquisition. Artificial intelligence can connect these layers, but simply concatenating high-dimensional features is unlikely to produce transportable clinical tools. This Mini Review examines how biologically informed machine learning, single-cell and spatial atlases, whole-omics response models, and validated tissue classifiers are reshaping prostate cancer risk stratification. We argue that the most useful architecture is a stage-aware system: ancestry-calibrated germline risk first modifies screening intensity; tissue or liquid-biopsy models then resolve tumor state; and longitudinal molecular measurements update risk under treatment. Evidence is strongest for polygenic enrichment of screening populations and for locked transcriptomic classifiers tested in randomized-trial cohorts. By contrast, many multi-omics models remain retrospective, dataset-dependent, and weakly calibrated across ancestry, disease stage, and specimen type. Progress requires explicit clinical decision points, external and prospective validation, uncertainty reporting, ancestry-aware calibration, and comparisons against simpler clinicopathological models. A unified framework should therefore treat artificial intelligence not as a single prognostic score, but as an auditable sequence of genomic updates that links inherited susceptibility to evolving tumor biology.

## 1 Introduction

Prostate cancer combines substantial inherited susceptibility with profound spatial, clonal, and treatment-induced heterogeneity; a prospective study of 203,691 Nordic twins estimated prostate cancer liability heritability at 57% (95% CI, 51%–63%) (Network CGAR, 2015). Its recurrent alterations span androgen-receptor signaling, ETS fusions, DNA-repair defects, PI3K pathway disruption, and lineage-plastic states (Mucci et al., 2016). Molecular taxonomies established seven alteration-defined primary-tumor subtypes: four defined by *ERG*, *ETV1*, *ETV4*, or *FLI1* fusions and three defined by *SPOP*, *FOXA1*, or *IDH1* mutations. Approximately 26% of primary tumors were not assigned to any of these seven subtypes, underscoring the incompleteness of this taxonomy (Wang et al., 2018). In advanced disease, integrative sequencing revealed convergent but clinically actionable changes in androgen-receptor and DNA-repair pathways (Abeshouse et al., 2015), while larger cohorts exposed a long tail of less frequent drivers (Robinson et al., 2015). These findings created a practical mismatch: inherited variants estimate the probability of developing cancer, whereas tumor assays describe the behavior of an already sampled lesion. Neither layer alone represents a patient’s changing risk trajectory.

This Mini Review asks how artificial intelligence (AI) can connect inherited susceptibility with tumor genomic state without collapsing biologically distinct signals into an opaque score. The focus is genomic risk stratification across screening, localized disease, and progression. We include machine-learning studies that integrate germline, genomic, epigenomic, transcriptomic, or liquid-biopsy features and emphasize externally validated clinical classifiers. Pure imaging AI, non-genomic clinical prediction, and unvalidated single-dataset signatures are outside the core scope. The central argument is that clinically credible integration should be stage-aware and sequential: germline information sets a prior risk; tumor molecular data update that risk after diagnosis; and longitudinal assays update it again during treatment (Figure 1).

**FIGURE 1:**
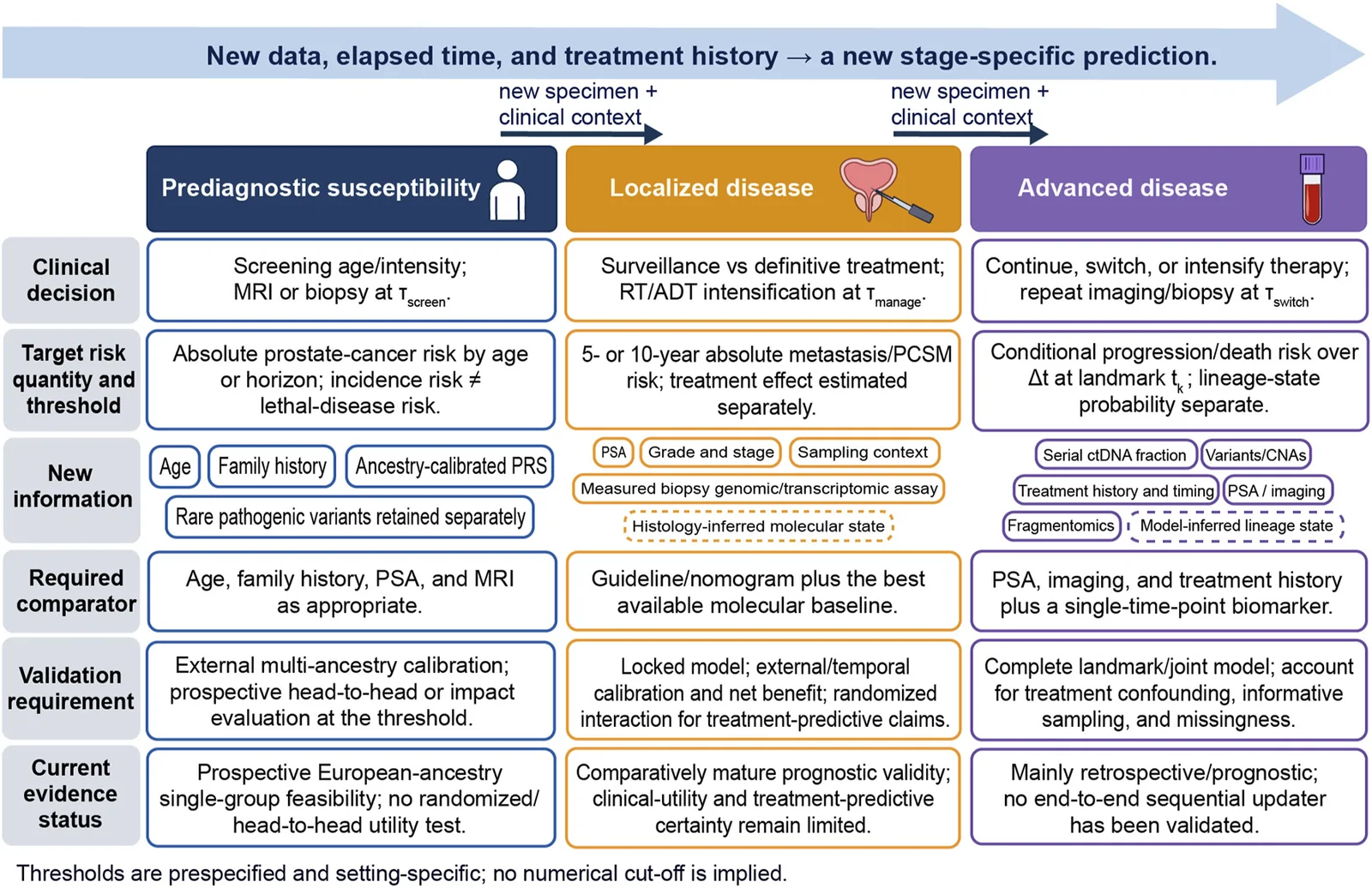
Decision-linked evidence map for stage-aware genomic risk updating in prostate cancer. Each column links a clinical stage to its decision, target risk quantity, new information, comparator, validation requirement, and current evidence. The symbols τ-*screen, τ*-manage, and τ-switch denote prespecified, setting-specific thresholds rather than proposed numerical cut-offs. Each transition creates a new prediction because the specimen, horizon, context, or treatment exposure has changed. Solid-outline input chips indicate directly measured or observed variables; dashed-outline chips indicate model-inferred molecular states that require concordance testing against the relevant assay. Evidence is asymmetric: PRS-guided screening has prospective single-group feasibility; localized classifiers have comparatively mature prognostic validity but limited utility or treatment-predictive certainty; and advanced longitudinal models remain mainly retrospective. Evidence labels reflect a narrative synthesis rather than formal GRADE assessment.

## 2 Review approach

For this targeted narrative Mini Review, PubMed was searched from database inception to 27 August 2026 using combinations of “prostate cancer” with “artificial intelligence,” “machine learning,” “polygenic risk score,” “genomic classifier,” “multi-omics,” “single-cell,” “spatial,” “circulating tumor DNA,” and “dynamic prediction.” Citation chaining identified additional primary and reporting-guideline papers. We prioritized human studies with prospective, external-cohort, randomized-trial-biospecimen, multi-ancestry, or biological validation. Selection was not systematic and was not performed in duplicate.

## 3 Tumor heterogeneity complicates multi-omics integration

Treatment-refractory metastatic tumors occupy AR-high, AR-low, amphicrine, double-negative, and neuroendocrine phenotypic states that are not captured by conventional grade alone (Armenia et al., 2018). Population context also matters. A genomic and epigenomic atlas of Asian prostate cancers identified differences in alteration frequencies and epigenetic programs relative to predominantly European-ancestry cohorts (Labrecque et al., 2019). At cellular resolution, joint single-cell multi-omic programs show that bulk measurements average clinically divergent populations (Li et al., 2020), while multifocal primary tumors can contain distinct transcriptomic programs within one prostate (Bian et al., 2024).

These observations constrain AI design. A model trained on one sampled focus may learn sampling geometry rather than patient-level aggressiveness. Molecular subtypes remain useful as intermediate biological representations, but should not be treated as immutable labels. AI is most valuable when it separates stable signal from specimen-, site-, and time-specific noise.

Heterogeneity also changes the prediction target. A label such as biochemical recurrence is assigned at the patient level, whereas genomic features are measured in a particular core, focus, metastatic site, or time point. The model may therefore be asked to infer a patient outcome from an incompletely observed tumor ecosystem. This mismatch is especially consequential for multifocal localized disease and lineage-plastic advanced disease (Armenia et al., 2018; Bian et al., 2024). Training sets should record sampling site, treatment exposure, tumor purity, and interval to outcome, and validation should test whether predictions remain stable across multiple regions or serial specimens. When such data are unavailable, uncertainty from sampling should remain visible rather than being absorbed into a falsely precise risk estimate.

## 4 Biologically informed and stage-aware integration

As summarized in the stage-by-decision evidence map (Figure 1), the information, prediction target, comparator, and validation requirement change across the disease course. Biologically informed neural networks offer one solution to the dimensionality problem. P-NET embedded known pathways into a sparse architecture, improving interpretability while identifying features distinguishing primary prostate cancer from metastatic castration-resistant prostate cancer (Salami et al., 2018). This approach contrasts with unconstrained feature concatenation, which can generate high apparent accuracy from small, internally correlated datasets. Reviews consistently identify generalizability, interpretability, heterogeneous endpoints, and weak external validation as principal barriers to AI genomic biomarkers (Elmarakeby et al., 2021). Even combined radiomic-genomic literature has been limited by small cohorts and inconsistent external testing (Bazarkin et al., 2024).

Recent models illustrate both opportunity and the remaining gap. A retrospective mCRPC response study used whole-genome sequencing from 155 men and matched whole-transcriptome sequencing from 113 men, with internal and external cohort evaluation (Castaldo et al., 2021). Blood genomic-lipidomic models were evaluated in 71 mHSPC and 144 mCRPC cases (de Jong et al., 2023). The NEP100 study integrated 19 bulk, 14 single-cell, and one spatial dataset with cellular experiments (Fang et al., 2023), whereas an endothelial signature used eight bulk and four single-cell cohorts plus functional testing (Shen et al., 2025). A trial-linked multiregion study analyzed 642 genomic samples from 114 men with a median 12-year follow-up (Lin et al., 2024). Histology-based deep learning predicted ERG status with an AUC of 0.86 in one external prostatectomy cohort and 0.80 in a biopsy cohort. A luminal-basal classifier was developed from 32,000 training and 68,547 evaluation profiles and explored in five clinical cohorts (Erak et al., 2023). Together, these studies show three integration routes: direct fusion of measured omics, biologically constrained latent representations, and cross-modal inference of an expensive molecular state from a cheaper modality.

These routes differ in missingness, interpretability, and the risk of substituting an inferred state for a measured assay. A predicted genomic state should not automatically replace a clinically validated assay; the intended decision and acceptable error must be specified before model construction (Fernandez-Mateos et al., 2024).

Integration can occur at input, representation, or decision levels. Early fusion is vulnerable to differences in scale, sparsity, and missingness; intermediate fusion can impose biological constraints; and late fusion can retain modality-specific models when an assay is unavailable. Ablation should compare each modality, the combined model, and a parsimonious clinical baseline. Without improved calibration or net benefit, complexity does not justify deployment (Salami et al., 2018; Castaldo et al., 2021; de Jong et al., 2023).

At prediagnosis, ancestry-calibrated PRS, rare variants, age, and family history estimate absolute risk. After diagnosis, grade, stage, and a locked tissue classifier update that risk; stage-specific molecular states such as CDK12 alterations can act as decision-linked modifiers (Weiner et al., 2023).

During advanced disease, risk must become longitudinal. In 738 plasma samples from 491 men with mCRPC, baseline ctDNA greater than 30% versus less than 2% was associated during first-line therapy with a fivefold higher risk of PSA progression (95% CI, 3.7–7.0) and a 5.6-fold higher risk of death (95% CI, 4.1–7.6) (Rescigno et al., 2021). These are prognostic associations, not evidence that ctDNA predicts differential treatment benefit. Fragmentomic features extracted from targeted panels add signal beyond variant calls alone (Fonseca et al., 2024). The resulting system is not one static model but a sequence of stage-specific dynamic predictions, each tied to a specimen, disease stage, treatment exposure, and decision window.

These safeguards build on existing consensus standards. TRIPOD + AI guides reporting of prediction models, PROBAST + AI assesses quality, risk of bias, and applicability, DECIDE-AI addresses early clinical evaluation, and CONSORT-AI addresses randomized trials of AI interventions (Helzer et al., 2023; Liu et al., 2020; Vasey et al., 2022; Collins et al., 2024). The additional problem considered here is sequential: a new prediction is generated when specimen type, disease stage, treatment exposure, or decision horizon changes, so the complete updating procedure—not only each component model—requires validation as a unit (Figure 2).

**FIGURE 2:**
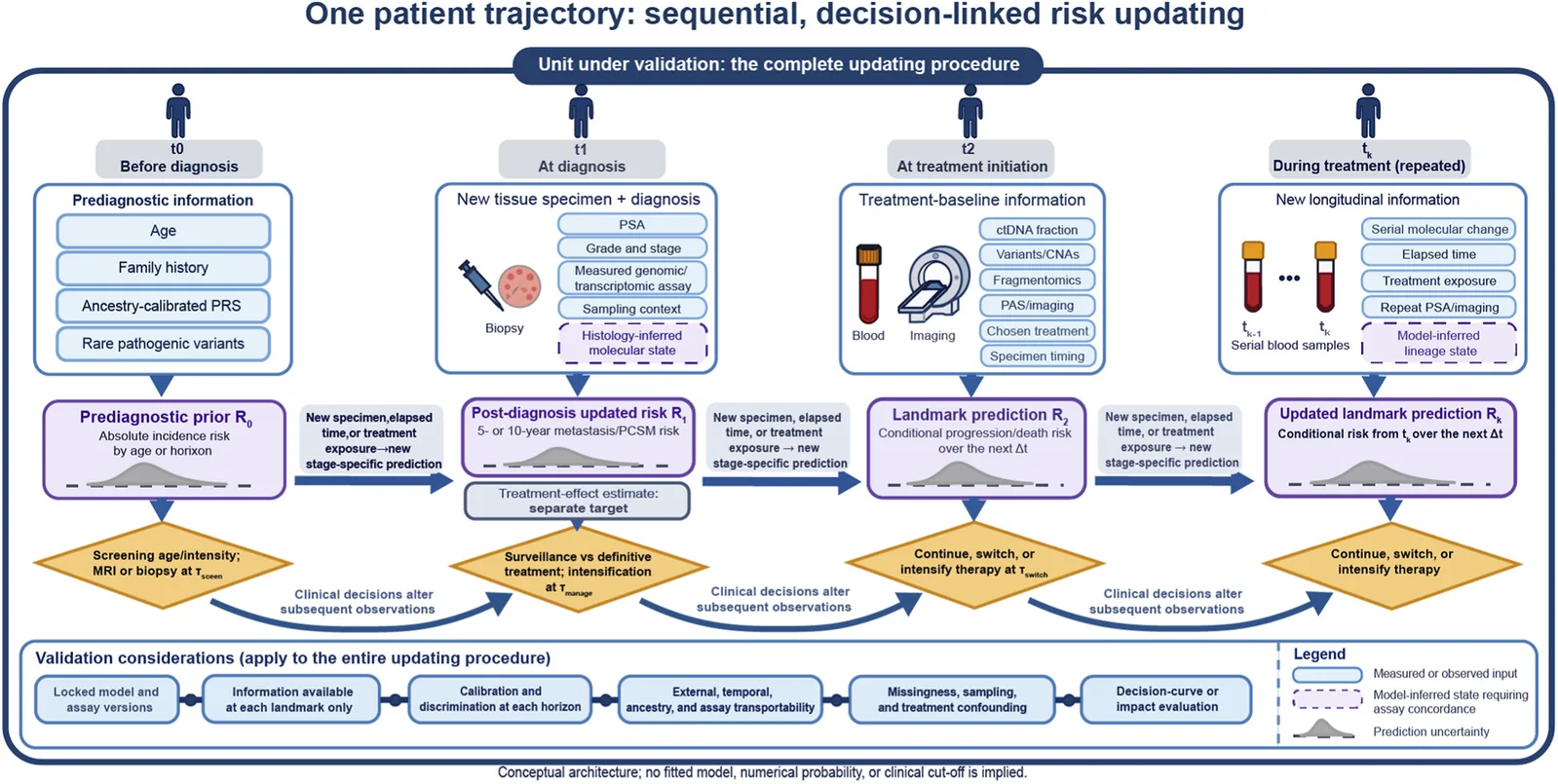
Sequential risk updating across specimens and landmark times in prostate cancer. A prediagnostic prior (R0) is estimated from information available before tissue acquisition, including age, family history, ancestry-calibrated polygenic risk, and rare pathogenic variants. At diagnosis, a new tissue specimen and clinicopathological context generate a post-diagnosis prediction (R1) for a stage-appropriate outcome and horizon; treatment-effect estimation remains a separate target. During treatment, serial plasma, imaging, elapsed time, and treatment history generate conditional landmark predictions (R2 to Rk) over a future interval Δt. Clinical decisions feed back into later observations, so post-treatment updates must address treatment confounding, informative sampling, and missingness. Solid-outline chips denote measured or observed inputs, dashed-outline chips denote model-inferred molecular states requiring assay-concordance testing. The outer envelope identifies the complete updating procedure as the unit requiring calibration, external and temporal validation, transportability assessment, and clinical-utility evaluation. The prior and updated-risk notation is conceptual and does not prescribe a fitted estimator or numerical clinical cut-off.

Three safeguards are necessary. First, the model must report uncertainty and abstain when input ancestry, specimen quality, or disease state lies outside the training domain. Second, validation must be separated by institution, calendar time, ancestry, and ideally assay platform; random train-test splits do not test transportability (Elmarakeby et al., 2021). Third, feature availability must be considered explicitly. A model that requires complete germline, tissue, spatial, and serial plasma data may exclude the patients most likely to benefit from better stratification.

Sequential updating also requires protection against feedback and treatment-confounding. A molecular measurement obtained after therapy reflects both tumor biology and prior clinical decisions; it cannot be interpreted as if it were a baseline prognostic marker. The update model should therefore include treatment history, specimen timing, and informative missingness, because patients undergo repeat biopsy or liquid-biopsy testing for clinical reasons. Dynamic performance should be assessed at prespecified landmark times, with predictions restricted to information available at each landmark. Landmarking and joint longitudinal-survival modelling provide formal implementations of this principle (Moons et al., 2025). The longitudinal value of ctDNA fraction and fragmentomics makes this design feasible (Rescigno et al., 2021; Fonseca et al., 2024), but an apparent improvement may still reflect greater disease burden rather than a treatment-specific mechanism. External validation should test the complete updating procedure, not only individual biomarkers.

## 5 Tissue molecular classifiers offer the closest clinical template

Localized prostate cancer provides the most mature clinical template for molecular prognostic validation, although incremental performance and clinical-utility evidence remain limited. Representative assays include Decipher, the Oncotype DX Genomic Prostate Score (GPS), Prolaris, and ProMark (Proust-Lima and Taylor, 2009; Cuzick et al., 2011; Klein et al., 2014). A systematic review of 39 studies involving more than 10,000 patients, 37 of them retrospective, found only modest improvements over standard clinical risk schemes on very low-to-low certainty of evidence; 22 studies evaluated Decipher, five GPS, and 14 Prolaris (Blume-Jensen et al., 2015). A second review found inconsistent effects on risk reclassification and treatment intensity (Boyer et al., 2025). The Decipher genomic classifier has been tested in archived specimens from randomized trials. In NRG/RTOG 0126, 215 quality-controlled cases with a median follow-up of 12.8 years showed that each 0.1-unit increase in the genomic-classifier score was associated with distant metastasis (sHR, 1.28; 95% CI, 1.06–1.55) and prostate cancer-specific mortality (sHR, 1.45; 95% CI, 1.20–1.76); 10-year distant-metastasis incidence was 4% in the low-score group and 16% in the high-score group (Tabriz et al., 2025). In the NRG/RTOG 9202, 9,413, and 9,902 meta-analysis, genomic-classifier scores were obtained for 385 specimens and 265 passed quality control; over a median 11-year follow-up, each 0.1-unit score increase was independently associated with distant metastasis (adjusted sHR, 1.22; 95% CI, 1.09–1.36), prostate cancer-specific mortality (adjusted sHR, 1.23; 95% CI, 1.09–1.39), and overall survival (adjusted HR, 1.12; 95% CI, 1.05–1.20) (Spratt et al., 2023). Across disease settings, a systematic review found consistent prognostic value but variable maturity of clinical-utility evidence (Nguyen et al., 2023). Independent validation in a high-risk post-prostatectomy cohort demonstrated incremental prognostic value (Jairath et al., 2021),and, in a retrospective cohort of 91 patients, higher genomic-classifier scores were associated with PSMA PET-detected nodal involvement (Karnes et al., 2018). Decipher has been evaluated in external post-prostatectomy cohorts and archived randomized-trial biospecimens, but several influential studies include assay-developer-affiliated authorship (Tabriz et al., 2025; Spratt et al., 2023; Jairath et al., 2021). This provenance does not negate the validation evidence; it makes transparent conflicts and fully independent replication especially important.

Clinical translation also depends on tissue adequacy, turnaround time, reimbursement, and whether testing changes management (Xu et al., 2019). Prognostic and treatment-predictive claims require different designs: prognosis estimates outcome under observed care, whereas prediction requires evidence of treatment-effect heterogeneity (Moschini et al., 2016).

The key lesson is not that one proprietary classifier should anchor all integration. It is that locked algorithms, prespecified endpoints, long follow-up, and randomized-trial specimens provide a validation template (Tabriz et al., 2025; Spratt et al., 2023; Nguyen et al., 2023). New multi-omics models should demonstrate calibration and net benefit at a defined decision threshold, then show that their added complexity changes care compared with the best available clinical and molecular baseline.

Discrimination alone is insufficient for that comparison. A higher concordance index or area under the receiver-operating-characteristic curve does not establish that a test moves patients across a treatment threshold correctly. Calibration should be examined at clinically relevant time horizons, and decision-curve analysis should compare the net benefit of testing with treat-all, treat-none, and guideline-based strategies (Ahmed et al., 2022). Trial-based classifier studies provide a stronger framework because outcomes, treatment, and follow-up were defined before molecular analysis (Tabriz et al., 2025; Spratt et al., 2023). Even then, retrospective use of archived tissue can establish clinical validity more readily than clinical utility. The decisive question is whether disclosure of the score improves a treatment decision and patient outcome, rather than whether the score remains statistically significant after adjustment (Nguyen et al., 2023; Xu et al., 2019; Moschini et al., 2016).

## 6 Polygenic risk sets the prediagnostic prior

Common germline variants can enrich screening populations before tumor tissue exists. In BARCODE1, 40,292 men were invited, 6,393 received a PRS, and 468 of the 745 men in the top decile underwent MRI and biopsy; 187 (40.0%) had prostate cancer and 103 (55.1% of cancers) had intermediate-or-higher-risk disease (Van Calster et al., 2018). These findings demonstrate prospective feasibility and enrichment, not comparative clinical utility: the study was single-group, restricted to men of European ancestry, and lacked a randomized or paired head-to-head initial-screen comparison with PSA or MRI (McHugh et al., 2025). A 269-variant trans-ancestry PRS was developed in 107,247 cases and 127,006 controls; the top versus middle decile odds ratio was 5.06 in European-ancestry men and 3.74 in African-ancestry men (Eklund et al., 2025). A polygenic hazard score was developed in 31,747 men and validated in 6,411 men; the hazard ratio for aggressive cancer was 2.9 (95% CI, 2.4–3.4) for the greater-than-98th versus 30th-70th percentiles (Conti et al., 2021). Independent validation of a 72-variant PRS yielded an AUC of 0.64 (95% CI, 0.62–0.66) (Se et al., 2018). Genome-wide Bayesian and penalized approaches can incorporate distributed subthreshold signal, but larger scores do not invariably improve prediction: a genome-wide PRS did not outperform PRS269 in African-ancestry (AUC, 0.656 vs. 0.679) or European-ancestry (0.844 vs. 0.845) samples (Black et al., 2020).

The distinction between relative and absolute risk is essential. A score’s distribution, calibration, and clinical threshold depend on the reference population; ancestry differences in epidemiology and genomics argue against a universal calibration function (Eklund et al., 2025; Darst et al., 2023).

Rare pathogenic variants address a related but different question. Germline testing and counselling identify variants with implications for relatives and, for selected genes, treatment, while their prevalence and interpretation vary by ancestry (Zhu et al., 2021; Russo and Giri, 2022). AI integration should retain separate channels for common-variant PRS and rare pathogenic variants rather than treating both as generic “germline features.”

A central controversy is whether susceptibility prediction can also identify biologically consequential cancer. Most prostate cancer PRSs were optimized for diagnosis, not prostate cancer-specific mortality or treatment benefit, so high inherited incidence risk should not be equated with an aggressive tumor phenotype (Conti et al., 2021; Se et al., 2018). In two population-based cohorts, an incidence-oriented PRS showed lower discrimination than midlife PSA for metastasis or prostate cancer-specific death (AUC, 0.63 vs. 0.78) and did not improve PSA-based prediction (Bataba et al., 2024). Associations with early onset, aggressiveness, or upgrading are informative but remain population- and endpoint-dependent. A stage-aware system should therefore use PRS primarily to modify the pretest probability and timing of screening, then allow measured tumor features to dominate postdiagnostic decisions. This separation reduces two opposing harms: intensified investigation of indolent disease solely because of high inherited risk, and false reassurance after a low PRS despite adverse tumor biology. It also makes calibration transparent because the germline and tumor components can be audited against different outcomes.

Equity is part of model validity, not an implementation afterthought. European-ancestry discovery cohorts still contribute disproportionate weight to many PRSs, and simple transfer of their effect sizes can misclassify risk in other populations (Eklund et al., 2025; Darst et al., 2023). Reporting a pooled discrimination statistic can hide this failure. BARCODE1 illustrates this limitation because its prospective screening cohort included only men of European ancestry; its thresholds and diagnostic yield therefore cannot be assumed to transport across ancestries (Van Calster et al., 2018; McHugh et al., 2025). Studies should provide ancestry-stratified calibration, sensitivity analyses for population structure, and absolute-risk estimates based on relevant incidence and competing-mortality rates. Local recalibration may improve predictions, but it cannot recover variants or gene-environment relationships absent from the development data.

## 7 Discussion

AI can connect inherited susceptibility and tumor state, but the evidence is asymmetrical. PRS is approaching prospective screening evaluation, and tissue transcriptomic classifiers have long-term trial-based validation. Multi-omics fusion is biologically richer but mostly retrospective. Multimodal deep-learning models trained on randomized phase III trial material demonstrate that locked AI can outperform conventional risk groups (Klein et al., 2022) and may predict clinically relevant outcomes for treatment personalization (Tward et al., 2024). The models reported by Esteva et al. (2022) and Tward et al. (2024) form part of the commercial Artera programme. According to NCCN Prostate Cancer version 5.2026, the ArteraAI Prostate Test is included as an option for localized disease (Spratt et al., 2026). Guideline inclusion does not remove the need for transparent conflict-of-interest reporting and independent external replication of the locked model. These histology-and-clinical models therefore define a relevant comparator: genomic inputs should be added only when they improve calibration or net benefit for a prespecified decision sufficiently to justify assay cost and missingness (Boyer et al., 2025; Ahmed et al., 2022).

As summarized in Figure 1, the proposed framework asks what information is available at each stage, which uncertainty is being reduced, what comparator is relevant, and what action follows. These requirements become more stringent with germline data because ancestry confounding, familial implications, and inequitable access can turn technical accuracy into clinical harm.

Several research priorities follow. Prospective studies should test whether PRS-guided screening followed by tumor molecular classification reduces overdiagnosis without missing aggressive disease (Van Calster et al., 2018). Models should compare sequential updating with simple side-by-side scores and report calibration, decision curves, subgroup performance, and failure modes. Federated or privacy-preserving analyses may improve cohort diversity, but harmonized phenotypes and assay quality remain prerequisites. Cross-modal models that infer molecular states from histology should quantify disagreement with measured assays and should not silently replace them (Fernandez-Mateos et al., 2024). Finally, clinical trials should evaluate whether a model identifies treatment-effect heterogeneity, not only prognosis (Klein et al., 2022; Tward et al., 2024).

This Mini Review is limited by its focused, narrative design and by rapid changes in the field. Because this was a targeted narrative review, no systematic search, formal risk-of-bias appraisal, or GRADE assessment was performed. It does not systematically grade every published signature, address image-only detection systems, or compare commercial assays economically. Nevertheless, the evidence supports a clear direction: prostate cancer risk should be represented as an auditable series of genomic updates from inherited susceptibility through evolving tumor state. AI will be clinically useful when it preserves these biological and temporal distinctions, reports when it is uncertain, and improves a specific decision beyond simpler alternatives.
